# L-Shaped Configuration of Multiple Magnet Ingestion: A Case Report

**DOI:** 10.34172/mejdd.2025.410

**Published:** 2025-01-31

**Authors:** Mehran Hakimzadeh, Mitra Ahmadi, Hazhir Javaherizadeh

**Affiliations:** ^1^Alimentary Tract Research Center, Clinical Sciences Research Institute, Imam Khomeini Hospital, Ahvaz Jundishapur University of Medical Sciences, Ahvaz, Iran

**Keywords:** Gastric, Magnets, Stomach

## Abstract

Multiple magnet ingestion is an urgent situation in pediatric gastroenterology that requires endoscopy or surgical intervention to remove the magnet. We report a case in which multiple magnets attract each other in a special geometric shape in the gastrointestinal tract of a 2-year-old boy.

## Introduction

 Foreign body ingestion is a potentially serious problem that peaks between 6 months to 3 years.^1^ Most of the foreign bodies were asymptomatic and require follow-up. However, some foreign bodies that cause intestinal obstruction require endoscopic or surgical procedures.^2^ Because multiple magnet ingestion may be asymptomatic, physicians must be aware of the possibility of multiple magnet ingestion because serious complications such as bowel perforation may occur.^3^

 There are several reports of multiple magnet ingestion in the literature. However, reports of the interesting shapes of multiple magnet ingestion are limited.^4^ Multiple magnets attract each other, but some geometric alignments may be of interest. Also, gastric perforation is a very important complication of multiple magnets ingestion and should be considered in children. Here in, L-shaped multiple magnet ingestion was reported.

## Case Report

**Figure 1:**
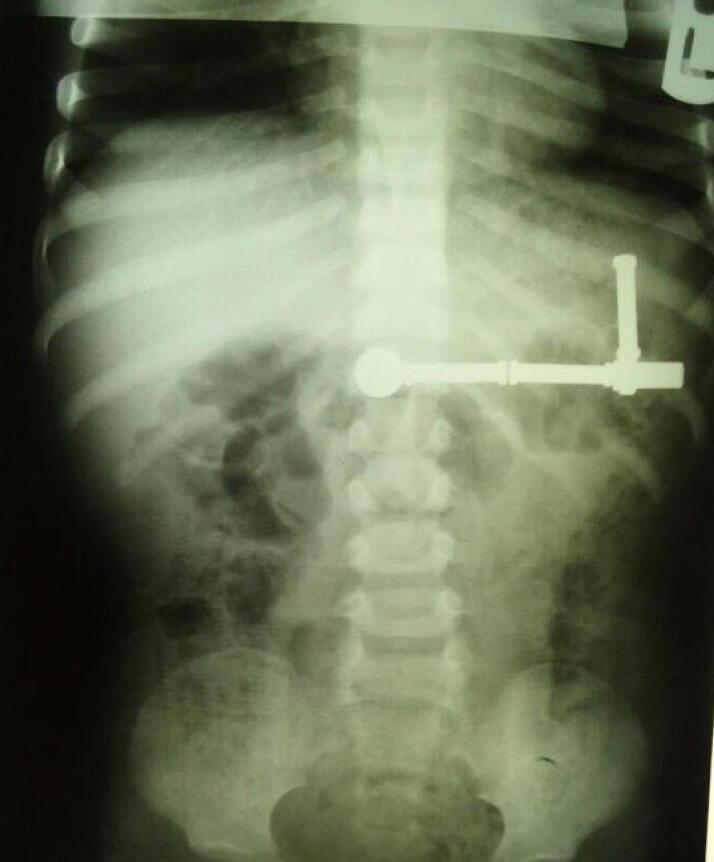
Multiple magnets in interesting alignment

**Figure 2:**
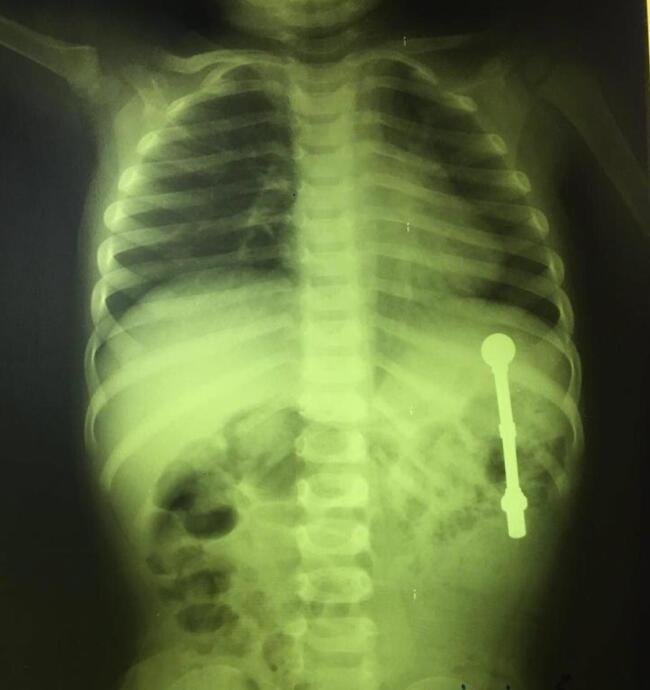
Magnets in the left upper quadrant

**Figure 3:**
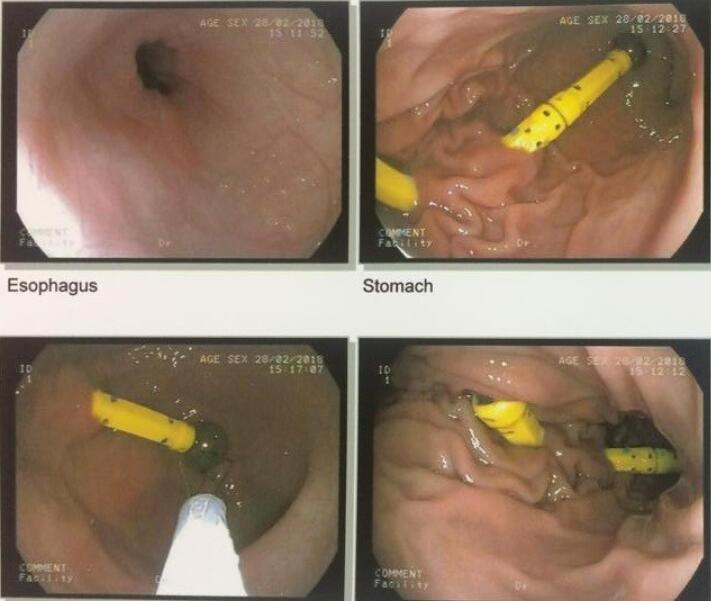
Multiple magnets penetrating gastric mucosa

 A 2-year-old Asian boy with multiple magnet ingestion was referred to our hospital. Magnet ingestion occurred during 24-48 hours before admission. Physical examination revealed no problem. Following abdominal radiography, multiple foreign bodies were seen in an interesting alignment (Figures 1 and 2). Evidence of gastric perforation was not seen in the radiographs, so an upper gastrointestinal endoscopy was done. Multiple magnets that penetrated gastric mucosa were seen (Figure 3). One magnet was removed using endoscopy with a snare and basket. Some magnets were inserted in the gastric tissue, so the patient was referred to a pediatric surgeon for removing other magnets.

## Discussion

 Single magnet ingestion causes no problem, but multiple magnet ingestion may cause some problems for children. They should be removed as soon as possible using an endoscopic procedure or surgery.

 In a recently published systematic review, 63.6% of children who ingested magnetic foreign bodies were male.^5^ Complications following multiple magnet ingestions were described in the literature.^6^ Bowel obstruction, bowel perforation,^7,8^ entero-enteric fistula,^9^ intestinal volvulus^10^ were reported. Intestinal volvulus and peroration were done following multiple magnet ingestion, as reported by Ilce and colleagues.^11^ Arslan and colleagues reported jejunoileal perforation following multiple magnet ingestion in a 3-year-old child.^12^ Also, De Raeymaeker et al reported a 2-year-old child with multiple magnet ingestion and intestinal perforation.^6^

 In this case, due to gastric perforation, surgical intervention was done. Surgical treatments of multiple magnet ingestion were reported in the literature.^13^

 For a diagnostic approach for children with suspected magnet ingestion, magnetic resonance imaging (MRI) is contraindicated. Perforation following MRI in children with unwitnessed magnet ingestion was reported.^14^ Recently, a high-frequency ultrasound was recommended to diagnose magnet ingestion.^15^

 In children without a history of magnet ingestion, magnet ingestion should be one of diagnoses.^16^ Peyron and co-workers reported a 6-year-old child who died after the onset of nausea and abdominal pain without a history of magnet ingestion. The autopsy showed diffused peritonitis and perforation of the transverse colon with multiple high-power magnets.^16^

## Conclusion

 Multiple magnet ingestion should be suspected in any child with a history of foreign body ingestion. Even without a history of foreign body ingestion, multiple magnet ingestion is one of the differential diagnoses in children who visit the hospital or clinics with gastrointestinal problems.^16^ Careful investigation is mandatory. Endoscopic and surgical intervention may be required.
